# 

**Published:** 1867-04

**Authors:** 


### Books and Pamphlets Received.

Obstetrics--The Science and the Art. By Charles D. Meigs, M. D. Lately Professor of Midwifery and Diseases of Women and Children, in the Jefferson Medical College at Philadelphia, and one of the physicians to the Lying-in-Department of the Pennsylvania Hospital; Member of the Society of Swedish Physicians at Stockholm, Corresponding Member of the Hunterian Society of London; Member of the American Philosophical Society; of the Natural Sciences of Philadelphia; of the American Medical Association, etc., etc. Fifth edition, revised. With one hundred and thirty illustrations. Philadelphia; Henry C. Lea, 1867. Breed, Lent & Co., Buffalo.

Researches upon "Spurious Vaccination," or the Abnormal Phenomena, accompanying and following Vaccination in the Confederate Army, during the recent American Civil War, 1861--1865. By Joseph Jones, M. D. Professor of Physiology and Pathology in the Medical Department of the University of Nashville, Tenn. From the Nashville Journal of Medicine and Surgery.

The American Conflict: A History of the Great Rebellion in the United States of America, 1861--1865; its Causes, Incidents and Results. Intended to exhibit especially its Moral and Political phases, with the Drift and Progress of American Opinion, respecting Human Slavery, from 1776 to the close of the War for the Union. By Horace Greeley. Illustrated by portraits on steel, of Generals, Statesmen and other eminent men; views of places of historical interest; maps; diagram of battle fields, naval actions, etc; from official sources. Vols. 1 and 2. For sale by agents. Lewis A. Filbert, 133 Elm street, Buffalo, N. Y. Price $10

An inquiry into the Origin of Modern Anæsthesia. By the Hon. Truman Smith, Member of the U. S. House of Representatives for the 26th, 27th, 28th, 29th and 30th Congress, and of the U. S. Senate for the 31st, 32d and 33d Congress. Hartford, Brown & Gross, 1867. Received from William Wood & Co., 61 Walker street, New York, through Breed, Lent & Co., Buffalo.

Practical Dissections. By Richard M. Hodges, M. D. Formerly Demonstrator of Anatomy in the Medical Department of the Harvard University. Second edition, thoroughly revised. Philadelphia, Henry C. Lea, 1867. Theo. Butler, 159 Main street.

The Half-Yearly Abstract of the Medical Sciences—being an analytical and critical digest of the principle of British and Continental Medical Works, published in the preceding six months. Vol. XLIV; July-December, 1866. Philadelphia: Henry C. Lea, 1867.

Digitalin--Its Chemical, Physiological and Therapeutic Action. An Essay to which was awarded a prize by the American Medical Association, May, 1866. By Samuel R. Percy, M. D. Professor of Materia Medica, New York Medical College, etc. Extracted from the Transactions of the American Medical Association.

### American Medical Association.

The Eighteenth Annual Meeting of this Association will be held at Cincinnati, Ohio, Tuesday, May 7th, 1867, at 11 A. M. Secretaries of all medical organizations are requested to forward lists of their delegates, as soon as elected, to the Permanent Secretary, Dr. W. B. Atkinson, Philadelphia, Pa.

